# The BioSynIRS trial: biologic versus synthetic mesh for multicompartment pelvic floor reconstruction

**DOI:** 10.1007/s10151-026-03404-7

**Published:** 2026-08-07

**Authors:** Claudia Rudroff, Luisa Halbe, Julia Schroer, Julius Ochel, Lars Daenenfaust, Axel Heidenreich, Sebastian Ludwig

**Affiliations:** 1 Department of Urology and Uro-Oncology, Interdisciplinary Continence and Pelvic Floor Center, University Hospital of Cologne and Medical Faculty Cologne, Kerpener Strasse 62, 50937 Cologne, Germany; 2 Clinic for General and Visceral Surgery, Evangelisches Klinikum Köln Weyertal GmbH, Academic Teaching Hospital of the University of Cologne, Cologne, Germany; 3 University of Cologne, Cologne, Germany; 4 Medical Faculty, University of Duesseldorf, Duesseldorf, Germany; 5 Institute for Health Economics and Clinical Epidemiology, University of Cologne, Cologne, Germany; 6 Department of Gynecology, Division of Urogynecology and Pelvic Reconstructive Surgery, University Hospital of Cologne and Medical Faculty Cologne, Cologne, Germany

**Keywords:** Biologic products, Feasibility study, Laparoscopy, Obstructed defecation syndrome, Pelvic organ prolapse, Surgical mesh

## Abstract

**Background:**

Selected patients with obstructed defecation syndrome (ODS) and concomitant pelvic organ prolapse (POP) may require laparoscopic resection rectopexy (L-RRP) with laparoscopic sacrocolpopexy (L-SCP). This trial compared the safety and efficacy of biologic versus synthetic mesh (BM or SM) in L-SCP.

**Methods:**

In this single-center randomized pilot trial, 30 women with severe ODS underwent combined L-RRP and L-SCP using either a BM or SM. The primary endpoint was the severe adverse event rate. Secondary endpoints included functional and anatomical outcomes related to ODS and POP. Follow-up was complete at 12 months for all patients.

**Results:**

The 30 patients (15 per group) had similar baseline characteristics, comparable frequency of additional surgical procedures, longer operative time in the SM group, lower pain in the BM group, similar length of hospital stay, comparable adverse event rates, and no mesh-related complications. Severe adverse events were reported by two patients (13%) in the BM group versus five (33%) in the SM group. Functional outcomes and POP-Q scores significantly improved in both groups (*p* < 0.01), urinary incontinence was unchanged, and there were three cases of ODS recurrence at 12 months. The main limitation of this study is its pilot design with a limited sample size and short follow-up, precluding definitive conclusions regarding long-term durability and rare adverse events.

**Conclusions:**

No significant differences in safety were detected between biologic and synthetic mesh; functional and anatomical outcomes improved similarly. Given the pilot design and limited sample size, these findings support the feasibility, but not equivalence or non-inferiority, of the biologic mesh. Adequately powered trials are required to confirm these findings and to evaluate the long-term durability of the biologic mesh.

**Trial registration:**

Trial registration at ClinicalTrials.gov (NCT06245577) and at the German Clinical Trials Register (DRKS00033079).

**Supplementary Information:**

The online version contains supplementary material available at 10.1007/s10151-026-03404-7.

## Introduction

Obstructive defecation syndrome (ODS) is frequently associated with pelvic organ prolapse (POP) and represents a complex manifestation of multicompartment pelvic floor dysfunction that substantially impairs quality of life [1–4]. In patients with persistent symptoms after failure of conservative treatment, surgical reconstruction of the pelvic floor is often required [5–9]. Interdisciplinary surgical strategies combining urogynecological and colorectal expertise allowing simultaneous correction of anterior, apical, and posterior compartments have gained acceptance and shown to be safe and effective, providing a standardized operative framework for the present comparative mesh-based study [10–15].

Synthetic meshes remain the standard material for sacrocolpopexy but are associated with relevant long-term complications, leading to regulatory warnings and persistent safety concerns, particularly in younger women [16–20]. Biologic meshes represent a resorbable alternative with favorable safety data in colorectal surgery [21–25]; their role in sacrocolpopexy, however, is unclear and it is currently not recommended in guidelines because of insufficient evidence [26]. Consequently, mesh selection in clinical practice is largely based on expert opinion and patient preference. 

This evidence gap provides the rationale for the present study comparing biologic and synthetic mesh materials in laparoscopic sacrocolpopexy within an established interdisciplinary surgical framework. Using a randomized controlled design, the study focuses on mesh-related safety and clinical outcomes. To our knowledge, this is the first randomized study to systematically evaluate mesh material in this clinical context, aiming to provide an evidence base for future trials and patient-centered decision-making.

## Methods

BioSynIRS (Biologic versus Synthetic mesh in Interdisciplinary Rectopexy and Sacropexy) was conducted as a single-center randomized controlled, single-blind, clinical pilot trial at the surgical department of the Evangelisches Klinikum Koeln Weyertal, Cologne, Germany.

After written informed consent, participants were enrolled and randomized to one of the two intervention groups prior to surgery. The experimental group underwent laparoscopic resection rectopexy (L-RRP) combined with laparoscopic sacrocolpopexy (L-SCP) using a biologic mesh (BM group), while the control group received a synthetic mesh (SM group). All randomized participants underwent a single surgical procedure. Participants were followed for 12 months after surgery. Outcome assessments were performed according to the predefined follow-up schedule as described below. Reporting here follows the extended CONSORT (Consolidated Standards of Reporting Trials) checklist for pilot and feasibility trials [27] (Table S1).

Women aged ≥ 18 years suffering from ODS and POP, requiring surgery, and suitable for laparoscopic surgery were assessed for eligibility. Planned pregnancy at or within 12 months after the study, previous apical support with mesh, and allergy to mesh materials were exclusion criteria. From this cohort those patients planned for L-RRP combined with L-SCP were included.

The study included patients scheduled for an interdisciplinary L-RRP combined with L-SCP. Urogynecological and surgical examinations, MRI defecography, abdominal CT scan, or MRI Sellink, a colon enema radiograph, and a routine review by the interdisciplinary pelvic organ board at our tertiary pelvic floor center were mandatory preoperatively.

For randomization, a list of computer-generated permuted block sizes of 4 and 6 was prepared. Enrolled patients were randomly assigned in a 1:1 ratio to one of the two groups. Allocation concealment was achieved by text messaging via mobile phone: After each enrollment, the study physician sent a participant-unique identifier and an independent person from outside the hospital replied by sending the group assignment.

Patients were masked to their assignment group. The masking of patients was maintained until their 12-month visit. In addition, physicians responsible for postoperative follow-up assessments at the study center and external physicians involved in subsequent patient care were blinded to treatment allocation. As a result of the nature of the intervention, blinding of the operating surgeons was not feasible.

All procedures were performed laparoscopically under general anesthesia with the patient in dorsal lithotomy position and 18° Trendelenburg as described previously [12, 28].

The rectum was fully mobilized to the pelvic floor by opening the retrorectal and rectovaginal spaces, followed by resection of the elongated sigmoid and anterior rectum. Intestinal continuity was restored by a stapled end-to-end colorectal anastomosis. Apical fixation of the middle pelvic compartment was performed with the mesh sutured to the vaginal vault, cervical stump, or dorsally to the cervix in cases of uterine preservation and anchored to the anterior longitudinal ligament at the sacral promontory with sutures. A suture rectopexy was then carried out by suturing the perirectal peritoneum to the sacral promontory. Depending on the mobility and anatomical configuration of the perirectal peritoneum, this rectopexy was performed unilaterally (left- or right-sided) or bilaterally, using one to three interrupted sutures per patient; suture laterality and number were recorded prospectively for each patient (Table 2). Non-resorbable sutures were used for mesh fixation at the sacral promontory and, where applicable, at the cervix, as the tensile strength of resorbable sutures is typically maintained for no longer than 150 days. This timeframe was considered insufficient to ensure durable scar formation and thereby safeguard the long-term reconstructive result.

The synthetic mesh material consisted of polyvinylidene-fluoride (DynaMesh VASA, FEG Textiltechnik GmbH Aachen, Germany). In case of biologic mesh, the Biodesign® Rectopexy Graft (Cook Biotech Incorporated, USA) was applied.

Baseline characteristics (e.g., age, body mass index (BMI), comorbidity assessed by American Society of Anesthesiologists (ASA) classification [29]) were recorded for all patients. Furthermore, data on medication, previous hysterectomy, and the diagnostic findings were collected. Operative data (operative time and length of hospital stay) and postoperative safety outcomes were documented prospectively. Postoperative follow-up comprised four visits: at hospital discharge, 4 weeks (± 7 days), 6 months (± 1 month), and 12 months (± 1 month) after surgery. At each visit, proctological and urogynecological examinations, clinical scores, and POP-Q staging were documented, and adverse events were systematically assessed. The total duration of participation for each patient was 12 months (± 1 month) from inclusion, with final outcome assessment at the last follow-up visit.

The primary outcome measure was postoperative safety, assessed by the rate and severity of adverse events using the Clavien–Dindo classification (CDC) [30] of morbidity and mortality at all follow-up visits, irrespective of causal relationship to the mesh or surgical procedure. This relatedness was not part of, and did not alter, the primary endpoint. After discharge, non-severe events (e.g., those treatable with drugs only) were only recorded if deemed (possibly) causally related to the initial therapy or the primary disease. For descriptive purposes only, all recorded adverse events were additionally classified post hoc as mesh-related, procedure-related, possibly procedure-related, or unrelated to the mesh or surgical procedure (Table 3).

Secondary outcome measures included functional and anatomical outcomes, evaluated with validated clinical scores. ODS symptoms were assessed using the Altomare score [31], bowel discomfort with the Rectal Toxicity score [32], fecal incontinence with the Wexner score [33], and urinary incontinence with the International Consultation on Incontinence Questionnaire (ICIQ, [34]); higher scores indicate greater symptom severity. Anatomical outcomes of POP were assessed by clinical examination using the Pelvic Organ Prolapse Quantification (POP-Q, [35]) system and Pain intensity by the numeric rating scale (NRS, [36]).

As this was a pilot study, no formal sample size calculation was performed. The prespecified sample size of 30 was chosen, because a recruitment period of about 12 months was deemed feasible [37]. Furthermore, a group size of 15 participants allows one to identify adverse events that might occur with a frequency of 20% or more.

Data were collected in a spreadsheet computer file, pseudonymized, and analyzed in a statistical software (PSPP V2.0). Demographic and clinical variables were compared between the two groups by applying either Student’s *t* test for continuous, Fisher’s exact test for dichotomous, or chi square test for other categorical variables. The Wilcoxon test was used to assess the statistical significance of changes over time, i.e. pre–post differences. A *p* value of < 0.05 was considered significant. Given the pilot nature of this trial, all statistical analyses are exploratory, and *p* values should be interpreted descriptively rather than as confirmatory evidence.

During the preparation of this work, the authors used ChatGPT (OpenAI) in order to assist with English language editing and improve clarity of expression, as the authors are not native English speakers. After using this tool, the authors reviewed and edited the content as needed and take full responsibility for the content of the publication.

## Results

**Fig. 1:**
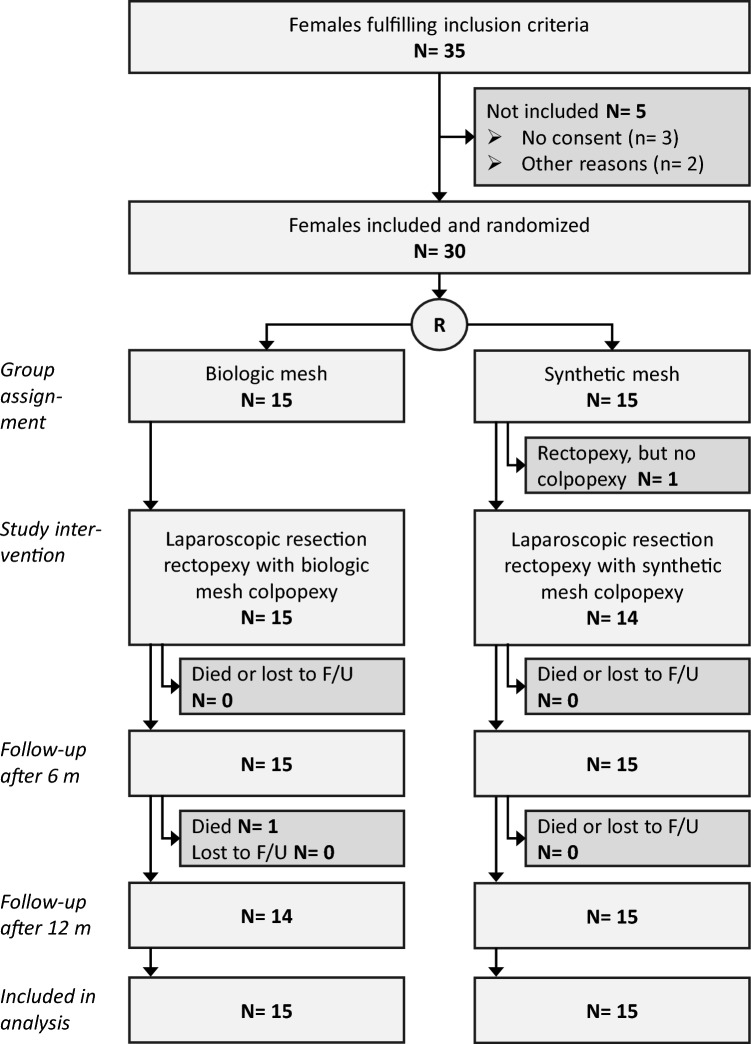
Trial flowsheet (according to CONSORT)

Between November 2023 and October 2024, a total of 150 women suffering from ODS and POP presented at our tertiary pelvic floor center for surgical therapy. Forty-five patients were scheduled for an interdisciplinary surgery approach and 35 planned for L-RRP combined with L-SCP fulfilling the inclusion criteria. Three patients were not included because of missing informed consent; two patients chose another hospital for the procedure (Fig. 1).

A total of 30 patients were included into two similar groups. In one patient, colpopexy could not be performed, as it was found to be already in place during surgery. The information on the previous surgery was incomplete. Following the intention-to-treat principle, this patient was included in all analyses, although no mesh was newly implanted. In a per-protocol sensitivity analysis excluding this patient from the sacrocolpopexy comparison, the conclusions regarding mesh-related safety and functional outcomes remained unchanged.

For the study cohort, mean age was 60.4 years (± 16.6, range 27 to 84) and mean BMI was 25.7 (± 4.5, range 18 to 37). The further details of the characteristics between the two groups showed no significant difference between the groups (Table 1). The sacrocolpopexy technique, including length and width of the mesh, and number of fixation sutures, did not differ between the groups (Table 1).

**Table 1:** Baseline characteristics of the study participant within the two groups

	Biologic mesh	Synthetic mesh	*p* value
No. of patients	15	15	–
Age, mean ± SD (range)	65.6 ± 15.1 (38–84)	55.3 ± 16.9 (27–77)	0.09
BMI, mean ± SD (range)	26.5 ± 4.8 (19.1–36.7)	25.0 ± 4.1 (18.0–32.0)	0.37
ASA risk class 1/2/3	2/6/7	2/9/4	0.49
Medication			
Anticoagulatory	3 (20%)	2 (13%)	0.67
Antihypertensive	7 (47%)	4 (27%)	0.29
Thyroid	7 (47%)	4 (27%)	0.29
Antidiabetic	1 (7%)	0	0.50
Diuretic	2 (13%)	2 (13%)	1.0
Previous hysterectomy	8 (53%)	9 (60%)	0.73
Incontinence			0.60
Stool and urinary	3 (20%)	3 (20%)	
Stool	2 (13%)	1 (7%)	
Urinary	3 (20%)	1 (7%)	
None	7 (47%)	10 (67%)	
Preoperative diagnostics			
Defecographic MRI	13 (87%)	15 (100%)	0.24
Defecography Conv	3 (20%)	0	0.11
Colon contrast	13 (87%)	14 (93%)	0.61
Abdominal CT	14 (93%)	12 (80%)	0.35
Colonic transit time (Hinton)	10 (67%)	12 (80%)	0.45
Magnetic resonance enteroclysis (Sellink)	0	3 (20%)	0.11
Diagnostic findings			
Rectocele	14 (93%)	10 (67%)	0.09
Intussusception	7 (47%)	12 (80%)	0.08
Enterocele	4 (27%)	4 (27%)	0.41
Rectal prolapse	4 (27%)	1 (7%)	0.19
Slow transit	3 (20%)	3 (20%)	1.0

*ASA* American Society of Anesthesiologists, *BMI*  body mass Index, *MRI* magnetic resonance imaging, *SD* standard deviation

In both groups additional procedures were performed, if necessary (Table 2). The duration of surgery in the SM group was significantly longer than in the BM group (*p* = 0.044; Table 2). After surgery, ten patients in the BM group and 12 patients in the SM group were treated for 1 day on an intensive care unit (ICU) unit (Table 2). In addition, pain intensity was slightly less severe in the biologic mesh group and distinct on day 1 after surgery (Table 2).

**Table 2:** Procedural, intraoperative and postoperative data (excluding complications) for the two study groups

	Biologic mesh	Synthetic mesh	*p* value*
No. of patients	15	15	–
Additional procedures			
Colporrhaphy	9 (60%)	11 (73%)	–
Adhesiolysis	6 (40%)	4 (27%)	–
Levator plasty	1 (7%)	1 (7%)	–
Other	3 (20%)	4 (27%)	–
None	1 (7%)	2 (13%)	–
Sacrocolpopexy technique			
Length of mesh (cm)	10.1 ± 1.1	11.5 ± 3.0**	0.11
Width of mesh (cm)	2.3 ± 0.6	2.1 ± 0.5**	0.56
No. of fixation sutures	4.9 ± 1.9	4.9 ± 1.1**	0.92
Resorbable sutures	2 (13%)	1 (7%)**	0.53
Rectopexy technique			
Suturing on right/left/both sides	8 (53%)/3 (20%)/4 (27%)	14 (93%)/0 (0%)/1 (7%)	0.04
No. of sutures	1.8 ± 0.7	1.6 ± 0.5	0.37
Resorbable sutures	3 (20%)	2 (13%)	0.67
Duration of surgery (mins)	294 ± 36 (range 240–350)	324 ± 45 (range 244–391)	0.06 (0.044)
Postoperative 1-day stay on ICU	10 (67%)	12 (80%)	0.45
Postoperative pain (NRS)			
Day 1	1.3 ± 1.7 (median 1, range 0–5)	2.6 ± 1.9 (median 2, range 0–7)	0.06 (0.053)
Day 3	1.8 ± 1.8 (median 1, range 0–6)	2.3 ± 1.6 (median 2, range 0–5)	0.46 (0.33)
At discharge	0.3 ± 0.8 (median 0, range 0–3)	1.1 ± 1.6 (median 0, range 0–5)	0.12 (0.11)
Length of hospital stay (days)	9.1 ± 2.6 (range 6–16)	8.0 ± 2.6 (range 3–15)	0.28

*Values in brackets are nonparametric test results

**Data missing for 1 patient who did not receive sacrocolpopexy

Exploratory analysis showed that pain on day 1 was associated with younger age (Table S2). Length of stay was similar between the two groups.

Both interventions were similarly safe. No mesh-related adverse events (e.g., mesh erosion, exposure, or infection) occurred in either group during the 12-month follow-up. The overall proportion of patients with adverse events did not differ between the groups, for any adverse event or for severe adverse events (*p* > 0.99 and *p* = 0.39 respectively, Table 3). The all-cause adverse-event counts included several events considered unrelated to the mesh or surgical procedure (e.g., COVID-19 infection, a fall during rehabilitation, COPD-related death, biliary pancreatitis, vulvar carcinoma with radiation skin damage). When these unrelated events were excluded, procedure-related or possibly procedure-related adverse events were less frequent and likewise did not differ significantly between groups (Table 3).

**Table 3:** Adverse events for both groups by the time of follow-up

	Biologic mesh	Synthetic mesh	*p* value*
Patients with			
No adverse event	7 (47%)	6 (40%)	
Any adverse event	8 (53%)	9 (60%)	> 0.99
Severe adverse event	2 (13%)	5 (33%)	0.39
Total number of adverse events (all causes)	11 (2 severe)	11 (5 severe)	
Adverse events in postoperative period	Cardiac decompensation, corona virus infection^†^, 2 × delayed bowel function	Rectal bleeding, delayed bowel function	
Adverse events after discharge until 1-month visit		Subileus, biliary pancreatitis*^†^, rectal bleeding*	
Adverse events between 1-month and 6-month visit	Fall during rehabilitation,^†^ subileus	Abdominal pain, adhesions*	
Adverse events between 6-month and 12-month visit	Overflow bladder, death due to COPD*^†^, urinary incontinence, irritable bowel syndrome, ODS recurrence*	Anastomotic insufficiency*, ODS recurrence, recurrent POP, vulva carcinoma with radiation damage to skin*^†^	
Procedure-related or possibly procedure-related	8 (1 severe)	9 (3 severe)	
Mesh-related	None	None	
Unrelated to mesh or procedure	3 (1 severe)	2 (2 severe)	

The primary endpoint (severe adverse event rate) is shown as a separate row; the rows below it present a post hoc descriptive breakdown of all recorded adverse events by causal relatedness; they do not redefine the primary endpoint

*These were severe adverse events (i.e., grade III or higher according to the Clavien–Dindo classification)

^†^Events were judged unrelated to the mesh or surgical procedure (corona virus infection, fall during rehabilitation, COPD-related death, biliary pancreatitis, vulva carcinoma with radiation damage to skin); all remaining events were considered as procedure-related or possibly procedure-related

One patient died from COPD-related lung disease. After 1 year, three patients (one in the BM group and two in the SM group) suffered from ODS recurrence. In a single case, mild POP reappeared with no specific treatment required.

In the BM group, two of 15 patients (13%) were affected by severe adverse events, numerically fewer than in the SM group (five patients, 33%); this difference was not statistically significant (Table 3). The 1-year follow-up in the BM group took place on average 418 days (± 91) after surgery, which was similar to the SM group with 443 (± 107) days.

**Fig. 2:**
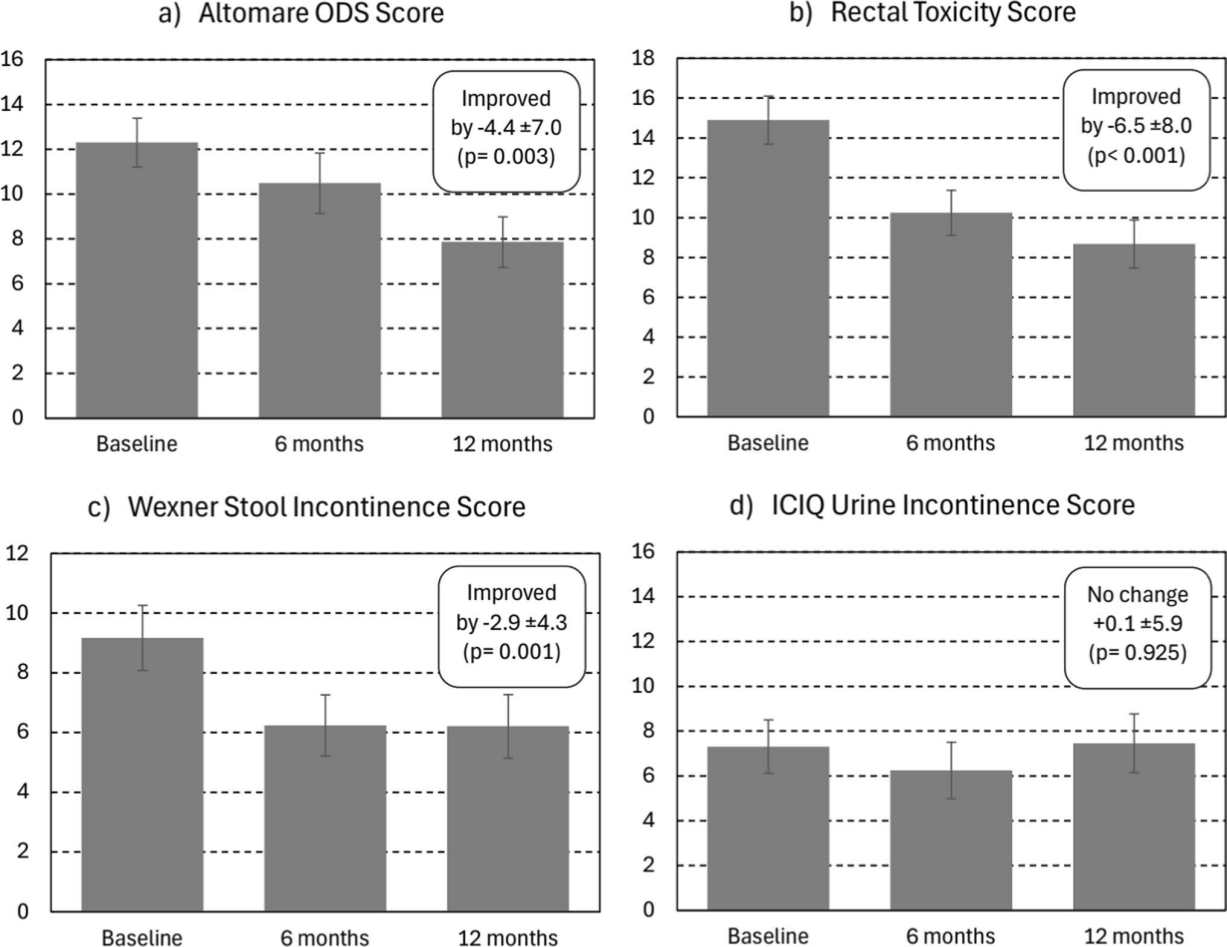
Change in clinical symptom scores, secondary endpoint of the study, before surgery and at 6 months and 12 months follow-up (whiskers indicate standard errors of the mean; *p* values are from Wilcoxon signed-rank tests comparing 12 months versus baseline)

Patient-reported outcome scores on ODS-related symptoms, bowel discomfort, and fecal incontinence were clearly improved by surgery (Fig. 2).

The severity of urinary incontinence symptoms, however, was mainly unchanged. The POP-Q score showed clear improvement; all women improved by at least one point. No between-group differences were noted in any of the scores (Table S3).

## Discussion

The key result of this pilot trial is that no statistically significant differences in safety were detected between biologic and synthetic mesh during 12 months of follow-up. No mesh-related adverse events occurred in either study group, and procedure-related adverse events were uncommon and did not differ significantly. The overall adverse-event counts included several clearly unrelated medical events and should not be interpreted as direct measures of comparative mesh safety. Severe adverse events were numerically fewer with biologic mesh (two versus five); however, the study was not powered to detect or rule out significant differences. Thus, study results should not be interpreted as evidence that biologic mesh reduces safety risk.

This finding is relevant given the ongoing concerns about synthetic mesh in pelvic floor surgery, including regulatory warnings, public debate, and patient apprehension [16, 38]. However, the principal unresolved question regarding biologic meshes is not perioperative safety but long-term durability after complete resorption of the graft—an issue this 12-month pilot trial was not designed to address. Within this limited scope, biologic mesh was successfully integrated into a standardized interdisciplinary framework without mesh-related complications. However, with only 15 patients per arm and no formal non-inferiority design, these findings do not establish equivalence or non-inferiority and should be regarded as preliminary and hypothesis-generating.

For functional outcomes, the interdisciplinary surgical approach resulted in significant and clinically meaningful improvements in ODS, bowel discomfort, fecal incontinence, and POP in both groups, sustained at 1 year, consistent with previously published results [12].

Crucially, no relevant differences were observed between the BM and SM groups for any of the functional outcome measures in the treatment of multicompartment pelvic floor dysfunction. This finding should not be interpreted as proof of functional equivalence between mesh types; rather, it indicates that no signal of inferiority was detected within the limits of this small pilot sample [39].

Urinary incontinence did not improve postoperatively, which is consistent with the literature, as the surgical strategy primarily addresses posterior and apical compartments [40]. Importantly, the absence of deterioration in urinary outcomes supports the overall safety and neutrality of the biologic mesh approach.

Early postoperative pain, a secondary endpoint, was lower with biologic mesh on day 1 but was primarily age-dependent, with younger patients reporting higher scores irrespective of mesh type, consistent with other reports [41]. As long-term surgical success depends on sustained, complication-free outcomes rather than early pain, this difference likely represents a minor perioperative benefit but has limited clinical significance.

A key clinical implication concerns patient choice. Increasing numbers of patients, particularly younger women, prefer to avoid permanent synthetic implants. The study’s data provide preliminary evidence that biologic mesh represents a feasible alternative that can be discussed with patients as part of informed shared decision-making, while transparently communicating that durability beyond 1 year remains uncertain.

The principal limitation is the pilot character, including limited sample size and follow-up duration. While sufficient to evaluate short-term safety and feasibility, the study was not designed to detect rare complications or long-term recurrence differences. Additionally, baseline imbalances may have influenced individual outcomes despite randomization. In particular, the biologic mesh group was on average approximately 10  years older than the synthetic mesh group (65.6 vs. 55.3 years). Although this difference did not reach statistical significance, the age gap may be of clinical relevance, and a confounding effect cannot be excluded in this setting. For postoperative pain, an exploratory subgroup analysis confirmed age as an independent predictor irrespective of mesh type. For complication rates and functional outcomes: age-adjusted analyses were not feasible with only two severe adverse events in the biologic mesh group and 15 patients per arm; any stratified or adjusted analysis would be statistically meaningless.

A further limitation concerns the concomitant procedures performed in both groups, most frequently colporrhaphy, and to a lesser extent adhesiolysis and levatorplasty (Table 2). Because these additional interventions were performed according to individual anatomical needs rather than mesh allocation, the observed functional improvements cannot be attributed solely to mesh type but rather to the interdisciplinary reconstructive concept as a whole. Suture rectopexy laterality also differed between groups (Table 2), reflecting surgeon judgment based on individual peritoneal mobility rather than a systematic protocol difference. While unlikely to explain the observed outcomes, this variation cannot be entirely excluded as a contributing factor given the small sample size.

This pilot study has fulfilled its purpose by demonstrating feasibility and an acceptable short-term safety profile for the use of biologic mesh in this setting. It provides methodological and clinical justification for a multicenter randomized trial with a longer follow-up of at least 36 months to assess durability, recurrence, and patient-reported outcomes in comparison to synthetic mesh material. Should future, adequately powered trials demonstrate non-inferiority, treatment decisions may increasingly be guided by informed patient preference, representing a step toward more patient-centered pelvic floor surgery.

In conclusion, no statistically significant differences in safety or short-term functional outcomes were detected between biologic and synthetic mesh used for apical fixation within an interdisciplinary surgical approach for multicompartment pelvic floor dysfunction. No mesh-related adverse events occurred in either group, and severe adverse events were numerically, but not significantly, fewer with biologic mesh; functional and anatomical results were similarly favorable at 12 months. Given the pilot design, small sample size, and short-term follow-up, these findings do not establish equivalence or non-inferiority and should be regarded as hypothesis-generating only. Larger, adequately powered randomized trials with extended follow-up are required to confirm these preliminary observations, and to evaluate the long-term durability of biologic mesh.

## Supplementary Information

Below is the link to the electronic supplementary material.

Supplementary file1 (PDF 197 KB)

## Data Availability

The datasets generated during and analyzed during the current study are available from the corresponding author on reasonable request.
