# Supplementary material for: The BioSynIRS trial: biologic versus synthetic mesh for multicompartment pelvic floor reconstruction
Source: Tech Coloproctol. 2026 Aug 7;30(1):129. doi: 10.1007/s10151-026-03404-7 (PMC13452879; doi:10.1007/s10151-026-03404-7)
Supplement: Supplementary file 1 [file 10151_2026_3404_MOESM1_ESM.pdf]

The BioSynIRS Trial: Biologic versus Synthetic Mesh in Interdisciplinary Resection Rectopexy with Sacrocolpopexy and for ODS with Pelvic Organ Prolapse

Claudia Rudroff[1,2]; Luisa Halbe[1]; Julia Schroer[3]; Julius Ochel[4]; Lars Daenenfaust[5]; Axel Heidenreich[1]; Sebastian Ludwig[6]

[1] Department of Urology and Uro-Oncology, Interdisciplinary Continence and Pelvic Floor Center, University Hospital of Cologne and Medical Faculty Cologne, Cologne, Germany

[2] Clinic for General and Visceral Surgery, Evangelisches Klinikum Köln Weyertal GmbH, Academic Teaching Hospital of the University of Cologne, Cologne, Germany

[3] University of Cologne, Cologne, Germany

[4] University of Duesseldorf, Medical Faculty, Duesseldorf, Germany

[5] Institute for Health Economics and Clinical Epidemiology, University of Cologne, Cologne, Germany

[6] Department of Obstetrics and Gynecology, Division of Urogynecology and Pelvic Reconstructive Surgery, University Hospital of Cologne and Medical Faculty Cologne, Cologne, Germany

<u>Corresponding author</u>

Claudia Rudroff, MD

Department of Urology an Uro-Oncology, Interdisciplinary Continence and Pelvic Floor Center, University Hospital of Cologne and Medical Faculty Cologne, Cologne, Germany

Email: claudia@rudroff.com;

Email institutional: claudia.rudroff2@uk-koeln.de

Supplementary file

Content:

- Table S1: CONSORT checklist for pilot trials
- Table S2: Exploratory analysis on the association between age and pain levels
- Table S3: Clinical and anatomical symptom score improvement

Table S1 CONSORT 2010 checklist of information to include when reporting a pilot or feasibility trial [1]

Section/Topic	Item No	Checklist item	Reported on page No
**Title and abstract**			
	1a	Identification as a pilot or feasibility randomised trial in the title	p. 1
	1b	Structured summary of pilot trial design, methods, results, and conclusions (for specific guidance see CONSORT abstract extension for pilot trials)	p. 2
**Introduction**			
Background and objectives	2a	Scientific background and explanation of rationale for future definitive trial, and reasons for randomised pilot trial	p. 3
	2b	Specific objectives or research questions for pilot trial	p. 3
**Methods**			
Trial design	3a	Description of pilot trial design (such as parallel, factorial) including allocation ratio	p. 6 and 7
	3b	Important changes to methods after pilot trial com-mencement (such as eligibility criteria), with reasons	Not applicable
Participants	4a	Eligibility criteria for participants	p. 6
	4b	Settings and locations where the data were collected	p. 6
	4c	How participants were identified and consented	p. 6
Interventions	5	The interventions for each group with sufficient details to allow replication, including how and when they were actually administered	p. 6
Outcomes	6a	Completely defined prespecified assessments or mea-surements to address each pilot trial objective specified in 2b, including how and when they were assessed	p. 6 and in trial registration
	6b	Any changes to pilot trial assessments or measurements after the pilot trial commenced, with reasons	Not applicable
	6c	If applicable, prespecified criteria used to judge whether, or how, to proceed with future definitive trial	Not applicable
Sample size	7a	Rationale for numbers in the pilot trial	p. 7
	7b	When applicable, explanation of any interim analyses and stopping guidelines	Not applicable
Randomisation:			
Sequence generation	8a	Method used to generate the random allocation sequence	p. 7
	8b	Type of randomisation(s); details of any restriction (such as blocking and block size)	p. 7
Allocation concealment mechanism	9	Mechanism used to implement the random allocation sequence (such as sequentially numbered containers), describing any steps taken to conceal the sequence until interventions were assigned	p. 5

Implementation	10	Who generated the random allocation sequence, who enrolled participants, and who assigned participants to interventions	p. 5
Blinding	11a	If done, who was blinded after assignment to interventions (for example, participants, care providers, those assessing outcomes) and how	p. 5
	11b	If relevant, description of the similarity of interventions	p. 4
Statistical methods	12	Methods used to address each pilot trial objective whether qualitative or quantitative	p. 5
**Results**			
Participant flow (a diagram is strongly re-commended)	13a	For each group, the numbers of participants who were approached and/or assessed for eligibility, randomly assigned, received intended treatment, and were assessed for each objective	Figure S1
	13b	For each group, losses and exclusions after randomisation, together with reasons	p. 6 and Figure S1
Recruitment	14a	Dates defining the periods of recruitment and follow-up	
	14b	Why the pilot trial ended or was stopped	Not applicable
Baseline data	15	A table showing baseline demographic and clinical characteristics for each group	Table 1
Numbers analysed	16	For each objective, number of participants (denominator) included in each analysis. If relevant, these numbers should be by randomised group	Table 3 and Figure S1
Outcomes and estimation	17	For each objective, results including expressions of uncertainty (such as 95% confidence interval) for any estimates. If relevant, these results should be by randomised group	p. 6
Ancillary analyses	18	Results of any other analyses performed that could be used to inform the future definitive trial	p. 6
Harms	19	All important harms or unintended effects in each group	Table
	19a	If relevant, other important unintended consequences	Table
**Discussion**			
Limitations	20	Pilot trial limitations, addressing sources of potential bias and remaining uncertainty about feasibility	p. 8
Generalisability	21	Generalisability (applicability) of pilot trial methods and findings to future definitive trial and other studies	p. 8
Interpretation	22	Interpretation consistent with pilot trial objectives and findings, balancing potential benefits and harms, and considering other relevant evidence	p. 8
	22a	Implications for progression from pilot to future definitive trial, including any proposed amendments	p. 8
**Other information**			
Registration	23	Registration number for pilot trial and name of trial registry	p. 4
Protocol	24	Where the pilot trial protocol can be accessed, if available	p. 10
Funding	25	Sources of funding and other support (such as supply of drugs), role of funders	p. 10
	26	Ethical approval or approval by research review committee, confirmed with reference number	p. 4

Table S2 Exploratory analysis on the association between patient age and postoperative pain (mean and standard deviation of NRS)

	Age under 50 years	Age between 50 and 69 years	Age 70 years or older	P value (ANOVA)
No. of patients	8	10	12	
Pain intensity				
On day 1	3.3 ±2.4	1.9 ±1.5	1.2, ±1.4	0.047
On day 3	2.4 ±1.3	2.7 ±1.9	1.3 ±1.5	0.11

Table S3 Clinical and anatomical symptom score improvement (ODS =obstructed defecation syndrome; internation clinical incontinence quantification= ICIQ; pelvic organ prolapse quantification POP-Q)

	**Biologic mesh**	**Synthetic mesh**	**p value***
Altomare ODS score			
Before surgery	13.3 ±5.7	11.3 ±6.3	0.39
6 months after surgery	11.6 ±8.3**	9.2 ±6.5	0.40
12 months after surgery	8.0 ±6.2	7.7 ±5.9	0.90
Rectal Toxicity score			
Before surgery	14.5 ±7.6	15.3 ±5.7	0.73
6 months after surgery	8.5 ±6.1**	11.2 ±5.9	0.25
12 months after surgery	8.4 ±7.0	8.9 ±5.9	0.84
Wexner fecal incontinence score			
Before surgery	9.5 ±6.4	8.9 ±5.7	0.79
6 months after surgery	5.9 ±6.3**	5.9 ±4.4	0.98
12 months after surgery	6.7 ±6.0	5.7 ±5.6	0.66
ICIQ urine incontinence score			
Before surgery	8.6 ±7.1	6.0 ±5.8	0.28
6 months after surgery	6.9 ±6.9**	4.7 ±5.8	0.37
12 months after surgery	8.0 ±8.2	6.9 ±6.1	0.69
POP-Q score (grades 0/I/II/III/IV)			
Before surgery	0 / 2 / 12 / 1 / 0	0 / 3 / 12 / 0 / 0	0.40
6 months after surgery	12 / 2 / 0 / 0 / 0	14 / 1 / 0 / 0 / 0	0.51
12 months after surgery	12 / 2 / 0 / 0 / 0	13 / 2 / 0 / 0 / 0	0.94

* Between-group differences were analyzed by Student's t test except for the POP-Q score, where chi square test for trend was applied.

** Data missing for 2 patient
